# Climate change and the emergence of Rift Valley fever virus: a pathological and environmental study of sheep jaundice in Egyptian slaughterhouses

**DOI:** 10.1186/s12917-025-05022-1

**Published:** 2025-09-17

**Authors:** Ashraf Kassem, Marwa S. Khattab, Elshaimaa Ismael, Aya M. Yassin, Dalia Hamza, Ahmed H. Osman

**Affiliations:** 1 Department of Pathology, Faculty of Veterinary Medicine, Cairo University, Giza, 12211 Egypt; 2 Department of Veterinary Hygiene and Management, Faculty of Veterinary Medicine, Cairo University, Giza, 12211 Egypt; 3 Department of Biochemistry and Molecular Biology, Faculty of Veterinary Medicine, Cairo University, Giza, 12211 Egypt; 4 Department of Zoonoses, Faculty of Veterinary Medicine, Cairo University, Giza, 12211 Egypt

**Keywords:** Climate change, Rift valley fever, Jaundice, Pathology, Egypt

## Abstract

**Background:**

The emergence of Rift Valley Fever Virus (RVFV) and its link to sheep jaundice in Egypt highlights the growing impact of climate change on the epidemiology of vector-borne diseases. Shifting climatic patterns, such as rising temperatures and altered rainfall, have expanded mosquito habitats, enhancing RVFV transmission risks. These environmental shifts create ideal breeding conditions for the mosquitoes, increasing virus’s transmission risk to both livestock and humans. Jaundice, a severe symptom resulting from RVFV infection, not only threatens livestock health but also poses significant economic challenges for farmers who rely heavily on their animals.

**Method:**

A descriptive case series study was performed to assess the impact of climate change on the total condemned sheep due to jaundice at Al-Basatin automated slaughterhouse. A total of 100 animals were examined from June to December 2024. Nineteen cases of jaundice were investigated to determine the cause. Gross examinations, histopathology, and immunohistochemical studies of caspase-3 and tumor necrosis factor alpha (TNFα), were conducted on various organs of jaundice affected cases. Frozen tissue samples were processed for molecular detection of RVFV, and determination of gene expression of heat shock 70KDa protein 1 A (HSP70) and bradykinin receptor B1 (BDKRB1). Malondialdehyde (MDA) and reduced glutathione (GSH), were analyzed to evaluate oxidative stress.

**Results:**

Gross examination of liver, kidneys, heart, and lungs of 19 jaundice affected sheep showed various lesions. Histopathological changes in 16 cases were indicative of RVF infection, while 3 cases were of unknown jaundice cause. RVF infection elevated immunoexpression of caspase-3 and TNFα. The presence of RVFV was confirmed in liver and kidney tissues. A significant correlation (*p* < 0.01) was observed between the occurrence of jaundice in sheep and extreme (THI > 25.6) to severe heat stress (HS) (THI = 23.2 to < 25.6). During the hot summer months, there was an increase in MDA, HSP70, and BDKRB1, and reduced GSH in liver and kidney.

**Conclusion:**

This study demonstrates a direct connection between climate change and the occurrence of RFV infection and jaundice in sheep. This relationship is likely linked to increased oxidative stress biomarkers in sheep and a weakened antioxidant defense system. These factors contribute to HS, triggering histopathological changes across all vital organs.

## Introduction

Climate change is a pressing global challenge with significant consequences for animal health and productivity [1]. Temperature variations, shifts in precipitation patterns, and extreme weather events all contribute to physiological stress, altered nutrition, and increased disease prevalence in animals [2]. Among these health concerns, jaundice poses a serious threat to sheep, particularly during the summer months, as it often results in the total condemnation of affected animals [3].

Climate change indirectly exacerbates jaundice in sheep through mechanisms such as heightened exposure to hepatotoxic agents, changes in the dynamics of vector-borne diseases, and environmental stressors that impair liver function [4]. The emergence and resurgence of infectious diseases, especially those transmitted by vectors like mosquitoes, are deeply influenced by climate change [5]. Several pathological causes of jaundice in sheep have been reported, including bacteria such as *Mycoplasma ovis*,* Anaplasma ovis*,* Leptospira sp.*,* and some Clostridium perfringens strains*, as well as others associated with the ingestion of toxic mineral compounds (copper poisoning), fungal toxins (aflatoxins), and plants (Allium sp. and Brassicaceae family) [3, 6]. One such disease, RVF is a leading cause of jaundice in sheep. This vector-borne illness is primarily spread by mosquito species such as Aedes and Culex and has been associated with severe outbreaks in regions like Africa and the Middle East [7].

The link between climate change and RVF is well-documented, as rising temperatures, altered rainfall patterns, and extreme weather directly impact mosquito populations and enhance virus transmission [8]. Climate change-driven increases in rainfall and flooding provide ideal breeding grounds for mosquitoes, triggering RVF outbreaks. In sheep, RVF manifests prominently as liver damage, which often progresses to jaundice [9]. The disease induces severe hepatocellular degeneration and necrosis, compromising the liver’s ability to process and eliminate bilirubin, a key factor in the development of jaundice [10, 11]. Our research delves into the intricate relationship between climate change, HS, and jaundice, and jaundice, focusing on understanding the pathogenesis of RVF in sheep.

## Materials and methods

### Animals’ population

This descriptive case series study was conducted on 100 sheep cases were examined after slaughter, which underwent condemnation in Al-Basatin automated slaughterhouses. The animals, aged 1–3 years and of various sexes, were investigated between June and December 2024. Nineteen cases that were condemned because of jaundice were investigated to determine its cause. The study protocol was reviewed and approved by the Institutional Animal Care and Use Committee of the Faculty of Veterinary Medicine, Cairo University, under approval number [Vet CU11052025115].

## Gross pathology

Nineteen condemned carcasses showed evident yellow discoloration of tissues consistent with jaundice during routine postmortem inspection and were thus selected for further analysis, and organs like the liver, kidneys, heart, and lungs were thoroughly examined postmortem to detect lesions associated with jaundice [12].

## Samples’ collection

Tissue samples from the liver, kidneys, heart, and lungs were collected from both control and affected animals and divided into two portions. The first portion was fixed in 10% neutral-buffered formalin for histopathological and immunohistochemical analyses. The second portion, obtained from six jaundiced sheep carcasses selected to represent different seasons (spring = 2, summer = 2, autumn = 2) and varying HS levels (extreme “THI > 25.6” = 4, normal “<22.2” = 2), was processed into homogenates for assessment of oxidative stress biomarkers, and evaluation of heat stress-related gene expression. Meanwhile, 19 sheep jaundice cases were used to detect RVFV [13].

## Histopathological examination

Tissue specimens from the liver, kidney, heart, and lungs were preserved in 10% neutral buffered formalin for histopathological examination and processed via paraffin embedding. 4 μm thick sections were prepared using a rotary microtome, stained with hematoxylin and eosin, and examined under a light digital microscope (Olympus xc30, Tokyo, Japan) [14]. Phloxine and Tartrazine stains were also used at 40x magnification to detect inclusion bodies following the method described in reference [15].

## Immunohistochemical examination

Following deparaffinization, rehydration, and antigen retrieval using citrate buffer (pH 6), primary antibodies against Caspase 3 (YPA 1086, China) and tumor necrosis factor alpha (TNF-α) (52B83, China) were employed. Following the manufacturer’s instructions, a secondary horseradish peroxidase (HRP)–labeled antibody was applied (Universal PolyHRP DAB kit for mouse and rabbit; Genemed, Sakura, Torrance, CA, USA). Hematoxylin was employed as the counterstain and diaminobenzidine as the substrate [16]. Negative control slides were stained with secondary antibodies only (not primary antibodies), and positive control in the targeted species. The Caspase 3 and TNF-α expressions were considered positive on an internal positive anatomical control.

### RVFV detection

Tissue samples from liver and kidneys were used for viral RNA extraction using the QIAamp Viral RNA Mini Kit and (its concentration and purity were evaluated using Thermo Scientific’s Nanodrop ND-1000 Spectrophotometer) according to the manufacturer’s instructions. For the molecular detection of RVF virus, a two-step RT-PCR was performed. In the first step, reverse transcription was carried out using the RevertAid First Strand cDNA Synthesis Kit (Thermo Fisher, USA). The reaction mixture consisted of 5 µL extracted RNA, 1 µL Oligo(dT) primers or random hexamers, 4 µL of 5× Reverse Transcription Buffer, 1 µL of Ribolock RNase inhibitor (20 U/µL), 2 µL of dNTP mix (10 mM each), and 1 µL of RevertAid Reverse Transcriptase (200 U/µL), with nuclease-free water added to a total volume of 20 µL. The reaction was incubated at 25 °C for 5 min for primer annealing, followed by 42 °C for 60 min for reverse transcription, and 70 °C for 5 min for enzyme inactivation.

In the second step, PCR amplification was performed targeting the Gc gene of the M segment using specific primers: Forward (5’-TGTGCACACGTATCTGCAGT-3’) and Reverse (5’-AAGAAGGCGGCATCACAAGA-3’). The reaction mixture contained 2 µL of synthesized cDNA, 10 µL of 2× PCR Master Mix (Thermo Fisher, USA), 1 µL of forward primer (10 µM), 1 µL of reverse primer (10 µM), and nuclease-free water to a final volume of 20 µL. The thermal cycling conditions included an initial denaturation at 95 °C for 5 min, followed by 35 cycles of 94 °C for 30 s (denaturation), 55 °C for 30 s (annealing), and 72 °C for 1 min (extension), with a final extension at 72 °C for 10 min. PCR products were analyzed using 1.5% agarose gel electrophoresis and visualized by ethidium bromide staining.

## Assessment of oxidative stress biomarkers

One hundred milligrams of liver and kidneys specimens were cut, collected in liquid nitrogen, and stored at −80 °C until further analysis. Liver and kidney tissues were homogenized in a 50 mM potassium phosphate buffer (pH 7.4), the mixture was centrifuged for 15 min at 4000 rpm. The supernatant was used for the measurement of reduced glutathione (GSH) and malondialdehyde (MDA) using commercial kits (Bio Diagnostic, Giza, Egypt) [17].

Briefly, GSH levels are determined by its reaction with 5,5′-dithiobis(2-nitrobenzoic acid) (DTNB), which reduces to form a yellow-colored product. The absorbance of this compound is measured at 405 nm, and the results are expressed in mg/gT [15]. According to [16], liver and kidney MDA concentrations (nM/gT) were measured as a marker of lipid peroxidation. The concentration of reactive thiobarbituric acid species in the supernatant was measured to ascertain the MDA content. At 534 nm, the absorbance of the resulting pink product was determined.

## Quantitative real-time RT-PCR analysis of BDKRB1 and HSP70

Total RNA was isolated from liver and kidney tissues using the Total RNA Purification Kit (Jena Bioscience, Germany, Cat. #PP-210 S), and its concentration and purity were evaluated using Thermo Scientific’s Nanodrop ND-1000 Spectrophotometer. Reverse transcription was performed by the RevertAid First Strand cDNA Synthesis Kit (Thermo Scientific, USA, Cat. #K1622), following the manufacturer’s guidelines.

Gene expression analysis was performed using a fluorescence-based real-time PCR method with β-actin (ACTB) as the internal reference gene. The reactions were set up with iQ SYBR^®^ Green Supermix (Bio-Rad, USA, Cat. #1708880). The primers.

listed in Table 1 were used for real-time RT-PCR. The PCR cycling conditions consisted of an initial denaturation at 95 °C for 3 min, followed by 40 cycles of denaturation (15 s at 95 °C), annealing (30 s at 60 °C), and extension (30 s at 72 °C). To ensure the specificity of the PCR products, melting curve analysis was conducted at the end of each reaction. All experiments were in triplicate, including a no-template negative control (NTC). The relative gene expression levels compared to the control were calculated using the 2^-ΔΔCT method [18].

**Table 1:** Sequence of primers used in quantitative real-time RT-PCR

Gene	Forward primer	Reverse primer	Amplicon size (bp)	Accession number
ACTB	5’-AAGTACCCCATTGAGCACGG-3’	5’-CATCTTCTCACGGTTGGCCT-3’	156	>*XM_060405599.1*
HSP70	5’-GATCAACGACGGAGACAAGC-3’	5’-GCTGCGAGTCGTTGAAGTAG-3’	182	>*NM_001267874.1*
BDKRB1	5’-CTCTTCGTGCTGTCCGTCTT-3’	5’-TGCTGATGAAGAGGTTGGCC-3’	208	*>XM_027957374.3*

*ACTB* Actin beta, *HSP70* heat shock 70KDa protein 1 A, *BDKRB1* bradykinin receptor B1

### Climate change evaluation

Environmental parameters, including ambient temperature, relative humidity, and precipitation, were obtained from the Egyptian Meteorological Authority (http://ema.gov.eg). Daily records corresponding to the sampling dates during the study period were used to calculate the heat stress indices, including the temperature–humidity index (THI) and the estimated Respiration Rate (RRest). Historical data covering the past five years (2019–2024) were collected at regular intervals.

### Heat stress indices calculation

The temperature–humidity index for sheep (THI_sheep_) was calculated using the standard formula [19]: THI_sheep_ = 𝐴𝑇 − {(0.31 − (0.31 × (𝑅𝐻 ∕ 100))) × (𝐴𝑇 − 14.4)} Where AT = Ambient temperature (°C); RH = Relative humidity (%). Marai et al. [20] categorized the heat stress for sheep according to the value of THI as: absence of HS (< 22.2 units); moderate HS (22.2 to < 23.2); severe HS (23.2 to < 25.6); and extreme HS (> 25.6). The estimated Respiration Rate (RRest) was calculated using the formula [21]: RRest = 5.1 t_db_ + 0.58 RH − 1.7 v_w_ + 0.039 r_s_ − 52.8. In which t_db_ is dry-bulb temperature, RH is relative humidity (%), v_w_ is wind speed (m/s), and r_s_ is solar radiation (W/m^2^). Four categories of RRest were established based on the original THI categories using the values of solar radiation of 800 W/m^2^ and a wind speed of 0 m/s. The categories for RR_est_ have the following thresholds: normal, 90; alert, 90–110; danger, 110–130; and emergency, ≥130.

### Statistical analysis

Results of the 19 jaundice cases were summarized as frequencies (%). The strength of the association between HS and the frequency of condemnation cases was tested by the Cramer’s *V* correlation; where values ≤ 0.20 indicate weak association, >0.20 to ≤ 0.60 indicate moderate association, and > 0.60 indicate strong association. A total of six jaundiced sheep carcasses were selected to represent different seasons (spring = 2, summer = 2, and autumn = 2) and varying levels of heat stress (extreme = 4, and normal HS = 2). The results for assessment of oxidative stress biomarkers, and evaluation of heat stress-related gene expression of the selected cases were presented as means and standard deviations (SD). Normality of the data was assessed using the Shapiro-Wilk test. Comparisons were conducted using statistical methods, including the independent sample *t-*test, one-way ANOVA, and Tukey’s post-hoc tests. The precipitation rate data were not normally distributed and were analyzed using the Kruskal-Wallis test. In addition, Eta squared (η²) was calculated to assess the effect size in one-way ANOVA comparisons across seasons (η² values: 0.01 = small, 0.06 = medium, ≥ 0.14 = large), and Cohen’s *d* was used to evaluate the effect size in independent sample *t-*test comparisons between the two HS-level groups (*d* values: 0.2 = small effect, 0.5 = medium effect, and 0.8 = large effect) [22], using effectsize [23] and effsize [24] R packages. Statistical analyses were performed using PASW Statistics Software (SPSS Inc., Chicago, IL, USA, Version 18.0), with a significance threshold set at *P* ≤ 0.05. Boxplots were generated using R (Version 4.4.3, R Foundation for Statistical Computing) using ggplot2 [25], ggpubr [26], and gridExtra [27] packages.

## Results

### Pathological examination

#### Gross pathology

**Fig. 1:**
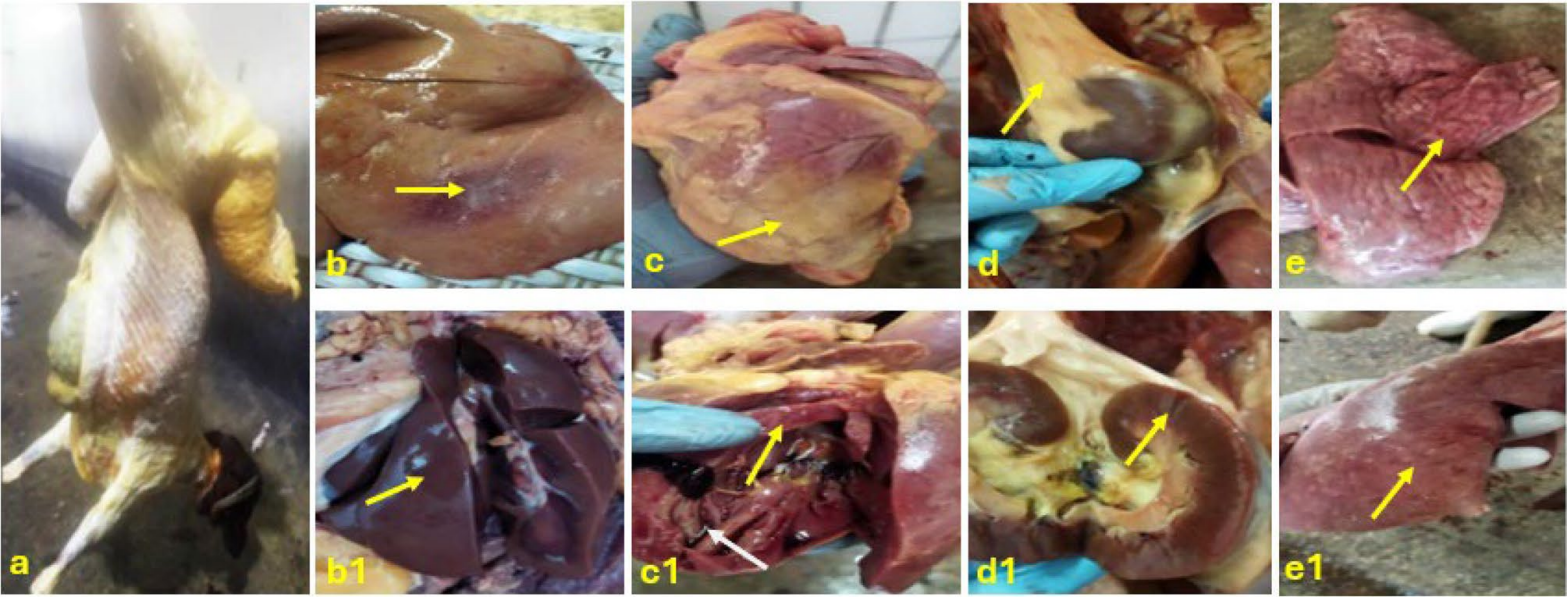
Macroscopic picture of jaundice in sheep carcass showing: (**a**) Total icteric carcass. (**b**) Greyish-white necrotic foci on liver surface (yellow arrow). (**b1**) Enlargement, soft and friable liver (yellow arrow). (**c**) Epicardial fat is yellowish (yellow arrow). (**c1**) White necrotic areas of myocardium (yellow arrow), and petechial hemorrhages on the endocardium (white arrow). (**d**) Yellowish perirenal fat (yellow arrow). (**d1**) Necrosis of renal cortex (yellow arrow). (**e and e1**) Congested and mottled lungs (yellow arrows)

The carcasses and internal organs of a total of 19 sheep condemned due to jaundice exhibited a yellowish discoloration of mucous and serous membranes (Fig. 1a). Greyish-white necrotic foci were visible on the liver’s surface 16 examined cases (Fig. 1b). The liver itself was moderately enlarged, soft, friable, and displayed a yellowish-brown coloration (Fig. 1b1). Additionally, in three emaciated cases, epicardial fat took on a yellowish hue and was accompanied by serous atrophy. The myocardium presented multifocal grayish-white necrotic regions, and the endocardium revealed petechial hemorrhages (Fig. 1c and c1). The perirenal fat and renal pelvis also appeared yellowish, while the kidneys showed subcapsular pale, anemic areas undergoing necrosis with a smooth surface (Fig. 1d and d1). Meanwhile, the affected lungs appeared congested, mottled, and enlarged (Fig. 1e and e1).

### Histopathological examination

**Fig. 2:**
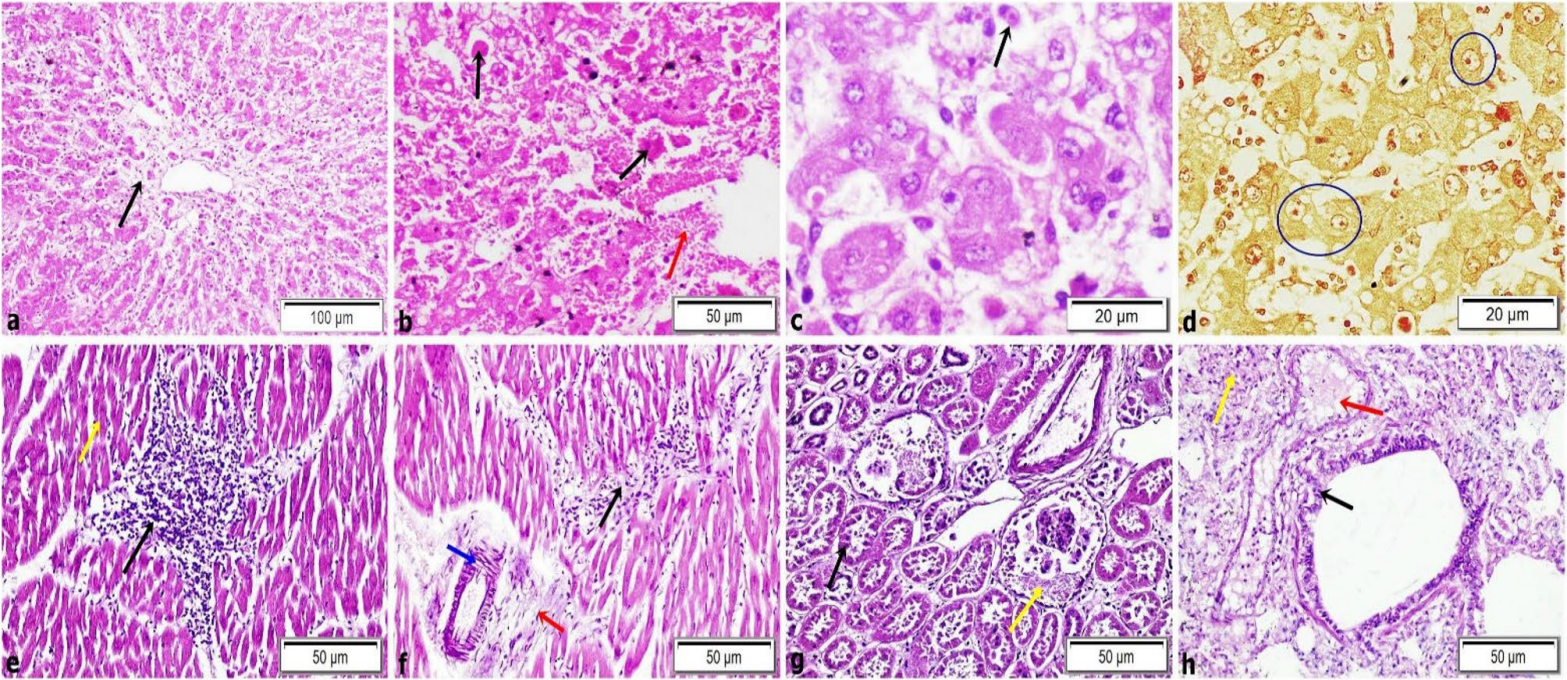
Histopathological examination of liver, heart, kidneys and lung tissues showing: **a** hepatic necrosis (black arrow). **b** Bilirubin cast (black arrow) and hepatic haemorrhage (red arrow). **c** Intranuclear inclusion bodies in hepatocytes (black arrow). **d** Red intranuclear inclusion bodies stained by Phloxine and Tartrazine staining (blue circle). **e** Coagulative necrosis of cardiomyocytes (yellow arrow), inflammatory cells infiltrations between muscle bundles and disorganization of muscle fibers (black arrow). **f** Perivascular edema (red arrow), perivascular cuffing by lymphocytes (black arrow) and dilatation of coronary branches (blue arrow). **g** Albuminous exudate in bowman’s capsule (yellow arrow) and tubular necrosis (black arrow). **h** Congested peribronchial blood vessels (red arrow), necrobiotic changes of bronchial epithelium (black arrow) and interstitial inflammation (yellow arrow)

The histopathological examination of hepatic tissue revealed fatty degeneration, vacuolation of hepatocyte and diffuse hepatic coagulative necrosis (Fig. 2a), hepatic hemorrhage characterized by extravasation of RBCs, hemosiderin pigment (dark yellow to brown granules), inflammatory cells infiltrations and bilirubin casts in-between disrupted hepatic cords (Fig. 2b). Intranuclear inclusion bodies were demonstrated in hepatocytes of 7 cases out of 16 total condemned jaundice (Fig. 2c and d). The cardiac tissue displayed severe alterations, including non-suppurative myocarditis, necrosis of cardiomyocytes, inflammatory cells infiltrations mainly lymphocytes and macrophages in-between muscle bundles, disorganization of muscle bundles (Fig. 2e). Perivascular edema and lymphocytic cuffing (Fig. 2f). Renal tissue exhibited eosinophilic proteaceous materials in Bowman’s space and tubular epithelial necrosis (Fig. 2g). Furthermore, the lungs tissue sections showed mild to moderate pathological changes represented by emphysema and interstitial pneumonia characterized by thickening of intra-alveolar septa by mononuclear cells as lymphocytes and macrophages (Fig. 2h). In addition, necrobiotic changes of bronchial epithelium and congested peribronchial blood vessels (Fig. 2h).

### Immunohistochemical examination

**Fig. 3:**
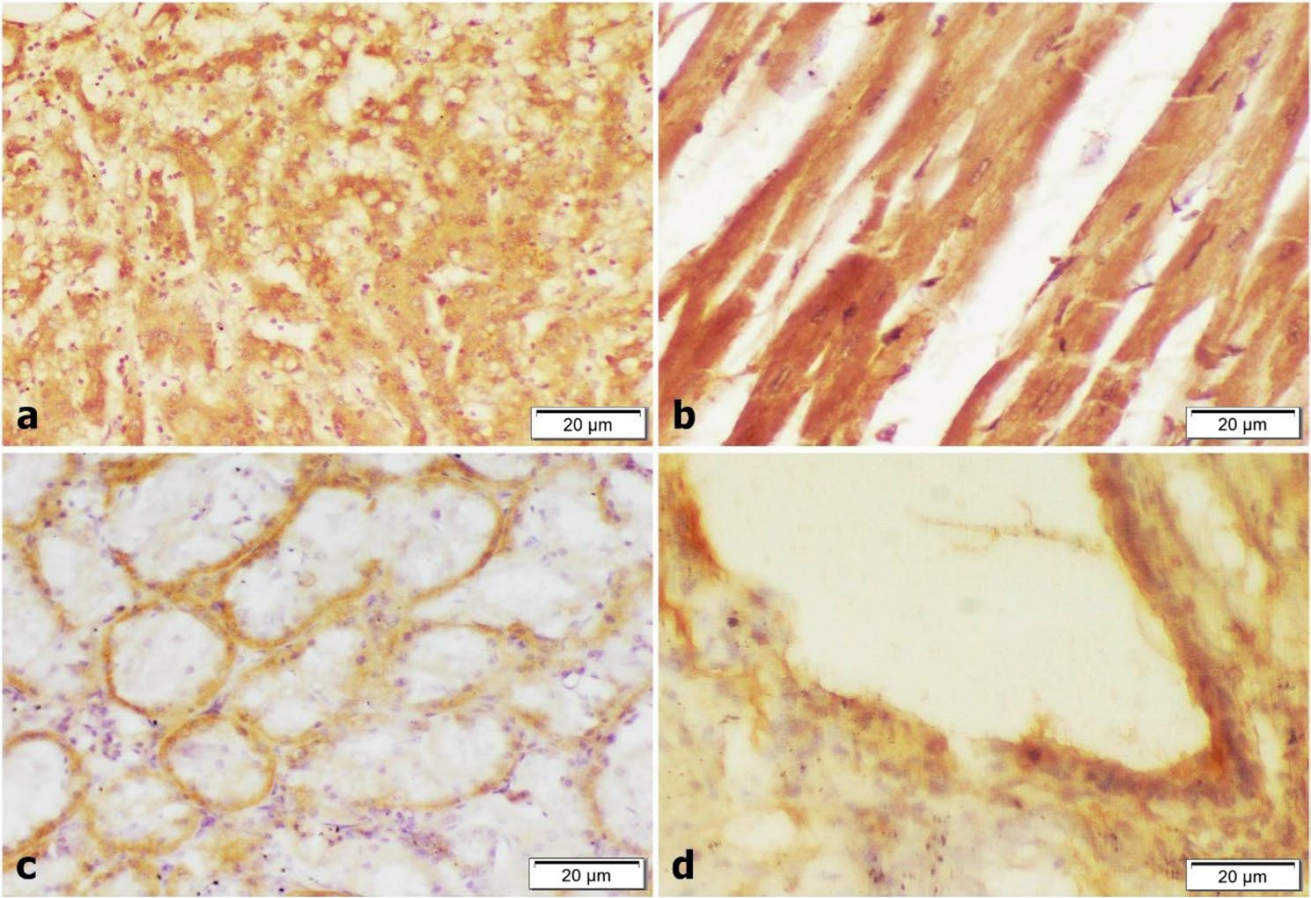
Immunohistochemical examination (Caspase-3) in liver, heart, kidney and lung (magnification 400X). **a** Increase cytoplasmic and\or nuclear of hepatocytes. **b** Degenerated cardiomyocytes showing positive expression. **c** Positive expression of degenerated renal cells. **d** Strong cytoplasmic staining of alveolar epithelium

Caspase-3 expression was expressed in the cytoplasm and/or nuclei of hepatocytes (Fig. 3a), necrosed cardiomyocytes (Fig. 3b), renal tubular cells (Fig. 3c) and degenerated alveolar epithelium (Fig. 3d).

**Fig. 4:**
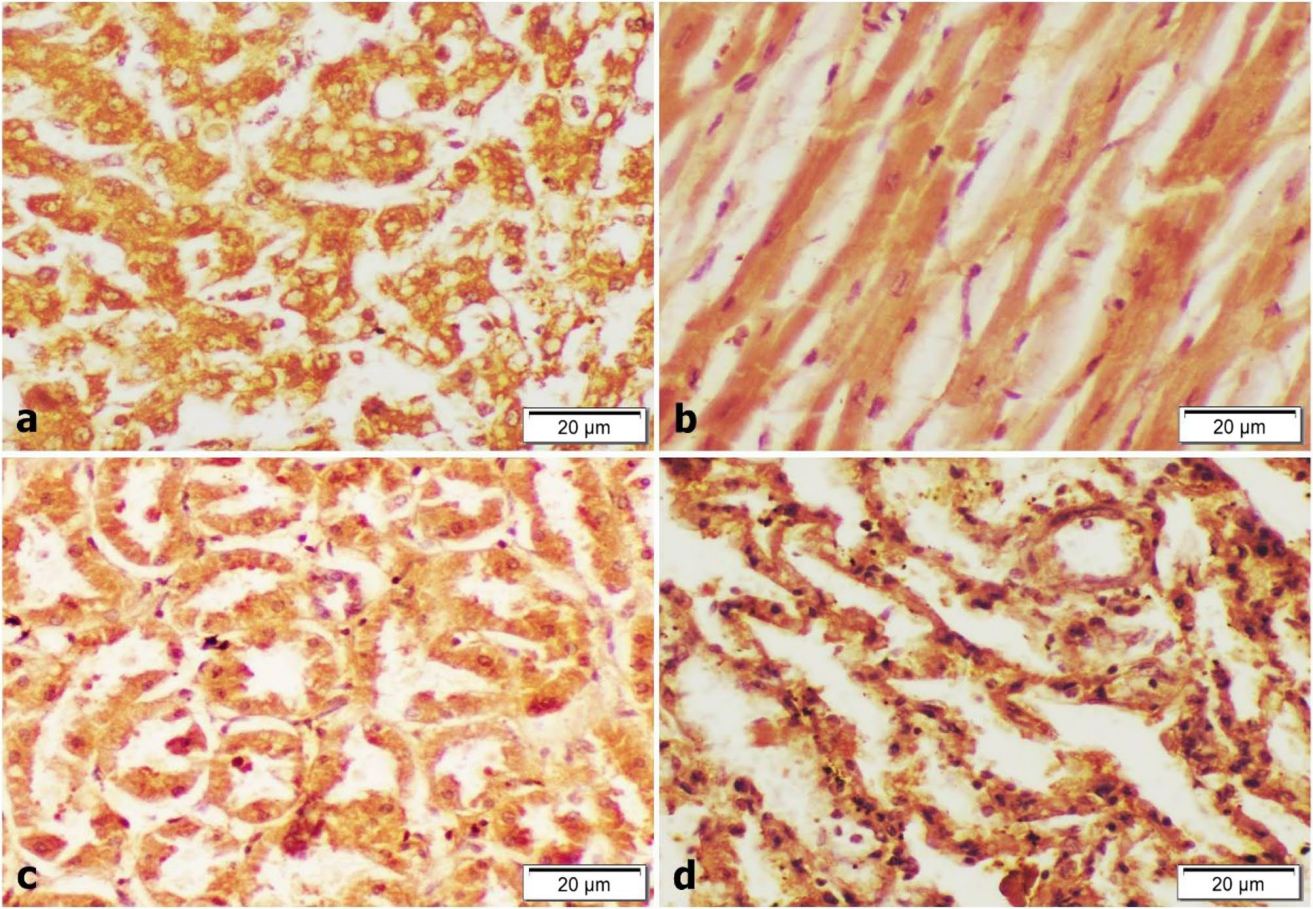
Immunohistochemical examination (TNF-α) in liver, heart, kidney and lung (magnification 400X). **a **and** b** Remarkable increase of TNF-α expression of the hepatocytes and cardiomyocytes. **c** Positive expression of tubular cells. **d** Moderate cytoplasmic staining of alveolar epithelium

Increased TNF-α expression was recorded in the cytoplasm of hepatocytes (Fig. 4a). strongly stained cells were found in cardiac tissue (Fig. 4b).The renal tubular cells showed significant increase TNF-α positive cells (Fig. 4c). Also, numerous strongly brown positively stained cells were demonstrated in bronchial epithelium (Fig. 4d).

### Gene detection of RVF

Results from our study showed molecular detection of m-RNAs in the studied genes, which detected RVF virus in the liver and kidney of 16 cases of totally condemned sheep.

### Expression of oxidative stress biomarkers in hepatic and renal tissue

**Fig. 5:**
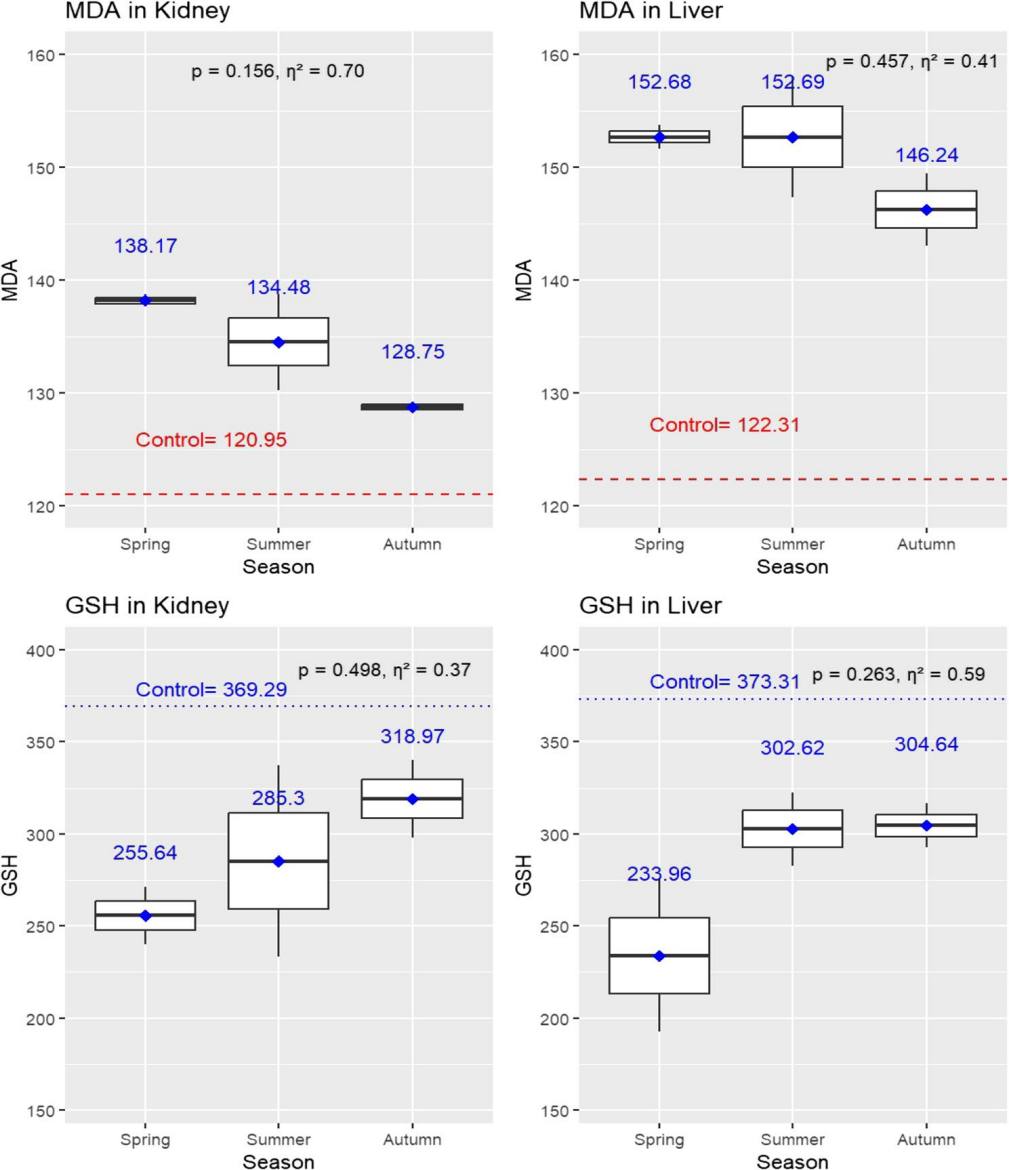
Seasonal variations in oxidative stress markers, malondialdehyde (MDA) and reduced glutathione (GSH), in kidney and liver tissues of jaundiced sheep carcasses (*n* = 6). Boxplots display oxidative stress levels across seasons, with mean values annotated above each box. Statistical comparisons were performed using one-way ANOVA with significance set at *p* ≤ 0.05. Effect size analysis (Eta squared η²) was included to assess the magnitude of differences, interpreted as follows: around 0.01 = small, 0.06 = medium, and ≥ 0.14 = large effect

There was a seasonal variation in MDA and GSH levels in kidney and liver tissues (Fig. 5). Regarding kidney tissues, MDA levels peaked during the spring (138.17 ± 0.76) and summer (134.48 ± 5.98) and declined in autumn (128.75 ± 0.67); (*p* = 0.156). Liver tissues displayed a similar trend, with MDA levels decreasing progressively across the seasons: spring (152.69 ± 1.52), summer (152.69 ± 7.60), and autumn (146.24 ± 4.56); (*p* = 0.457). On the other hand, in kidney tissues, GSH levels exhibited a progressive increase across the seasons, with values recording 255.64 ± 22.16 in spring, 285.30 ± 73.53 in summer, and 318.97 ± 29.70 in autumn (*p* = 0.498). In liver tissues, GSH levels displayed a different pattern. Levels measured 233.96 ± 58.45 in spring but peaked during summer (302.62 ± 28.28) and autumn (304.64 ± 16.96); (*p* = 0.263).

Although none of these seasonal differences were statistically significant, effect size analysis revealed large seasonal effects across all measured oxidative stress biomarkers. For MDA levels, large effects were observed in kidney (η² = 0.71) and liver tissues (η² = 0.41). Similarly, GSH levels showed large effect sizes in both liver (η² = 0.59) and kidney tissues (η² = 0.37). These findings suggest that seasonal variations may exert a meaningful biological impact on oxidative stress responses, highlighting the need for further research using larger cohorts to validate these observations (Fig. 5).

**Fig. 6:**
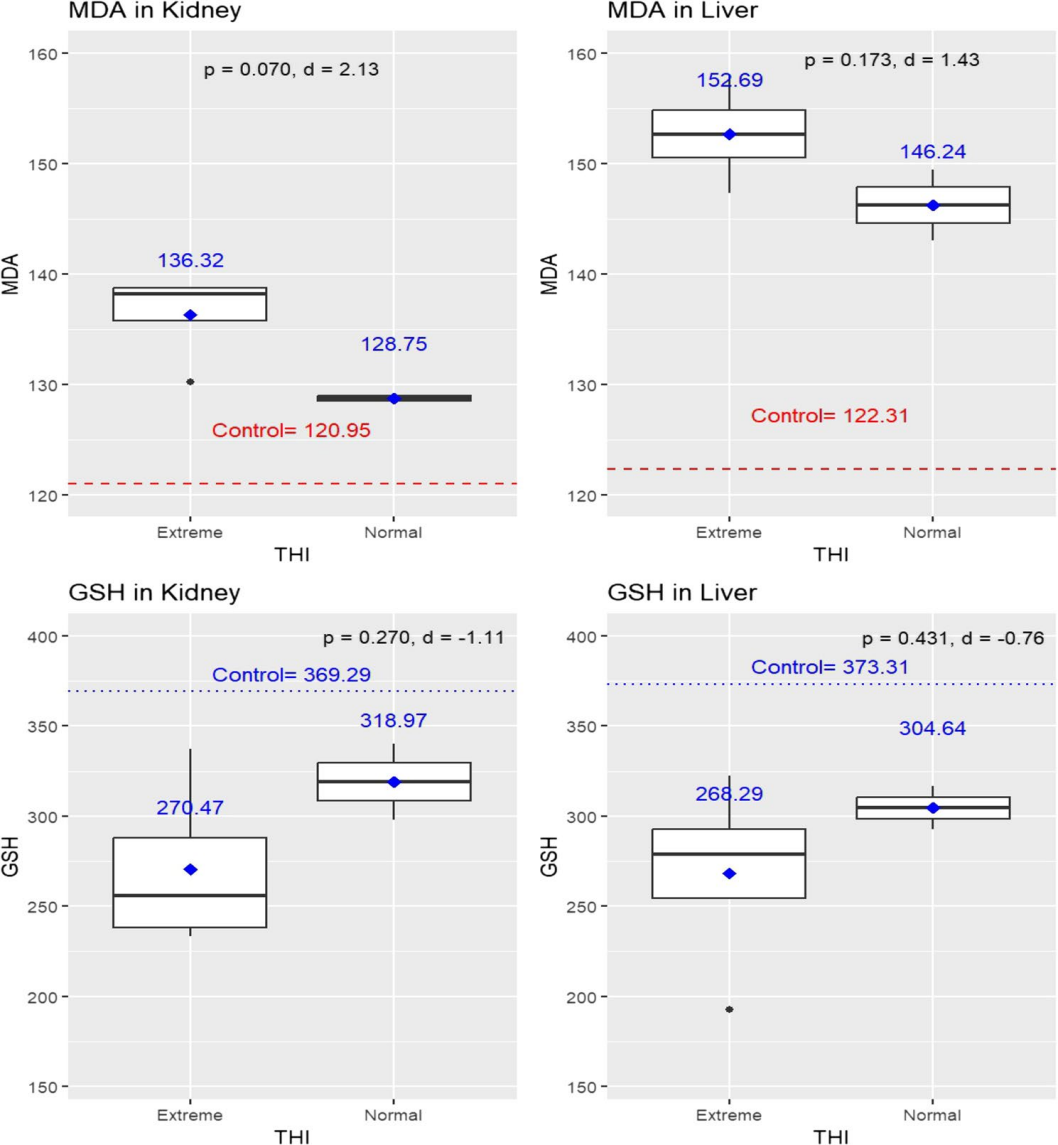
Oxidative stress markers, including Malondialdehyde (MDA) and Glutathione (GSH), observed in the kidneys and livers of jaundiced sheep carcasses (*n* = 6) under different heat stress conditions, as defined by the temperature-humidity index (THI). Boxplots show gene expression under extreme (THI > 25.6) and normal (THI < 22.2) conditions, with mean values displayed above each box. Statistical comparisons were performed using independent sample *t*-tests, with significance set at *p* ≤ 0.05. Effect size analysis (Cohen’s *d*) was included to assess the magnitude of differences, interpreted as follows: around 0.2 = small, 0.5 = medium, and ≥ 0.8 = large effect

Figure 6 shows HS-induced variations in MDA and GSH levels in kidney and liver tissues. Under extreme THI conditions, elevated MDA levels were observed in both the kidney (136.32 ± 4.08) and liver tissues (152.69 ± 4.48). Conversely, lower MDA values were associated with normal THI conditions, with levels recorded at 128.75 ± 0.67 for kidneys and 146.24 ± 4.56 for livers. However, these differences were not statistically significant for kidneys (*p* = 0.070) or liver tissues (*p* = 0.173). The seasonal and HS-induced variations in GSH levels in kidney and liver tissues are depicted. In contrast, HS, as indicated by THI, had a noticeable effect on GSH levels. Under extreme THI conditions, GSH levels were recorded at 270.47 ± 47.53 in kidney tissues and 268.29 ± 54.56 in liver tissues. By contrast, normal THI conditions were associated with higher GSH levels: 318.97 ± 29.70 in kidneys and 304.64 ± 16.96 in livers. However, these differences were also not statistically significant for kidneys (*p* = 0.270) or liver tissues (*p* = 0.431).

Despite the absence of statistically significant differences in MDA and GSH levels between extreme and normal THI conditions, effect size analysis using Cohen’s *d* indicated biologically relevant changes associated with heat stress. For MDA, large effect sizes were observed in both kidney (*d* = 2.13) and liver tissues (*d* = 1.43), suggesting a substantial increase in lipid peroxidation under extreme thermal conditions. Similarly, GSH levels showed a large negative effect size in kidney tissues (*d* = −1.11) and a medium negative effect in liver tissues (*d* = −0.76), indicating a marked reduction in antioxidant capacity with heat stress, particularly in renal tissues. These findings highlight that, even in the absence of statistical significance, the magnitude of change reflects a meaningful biological impact of heat stress on oxidative stress biomarkers (Fig. 6).

### Gene expression levels of the BDKR and HSP70 genes in the kidneys and livers of jaundiced sheep carcasses

**Fig. 7:**
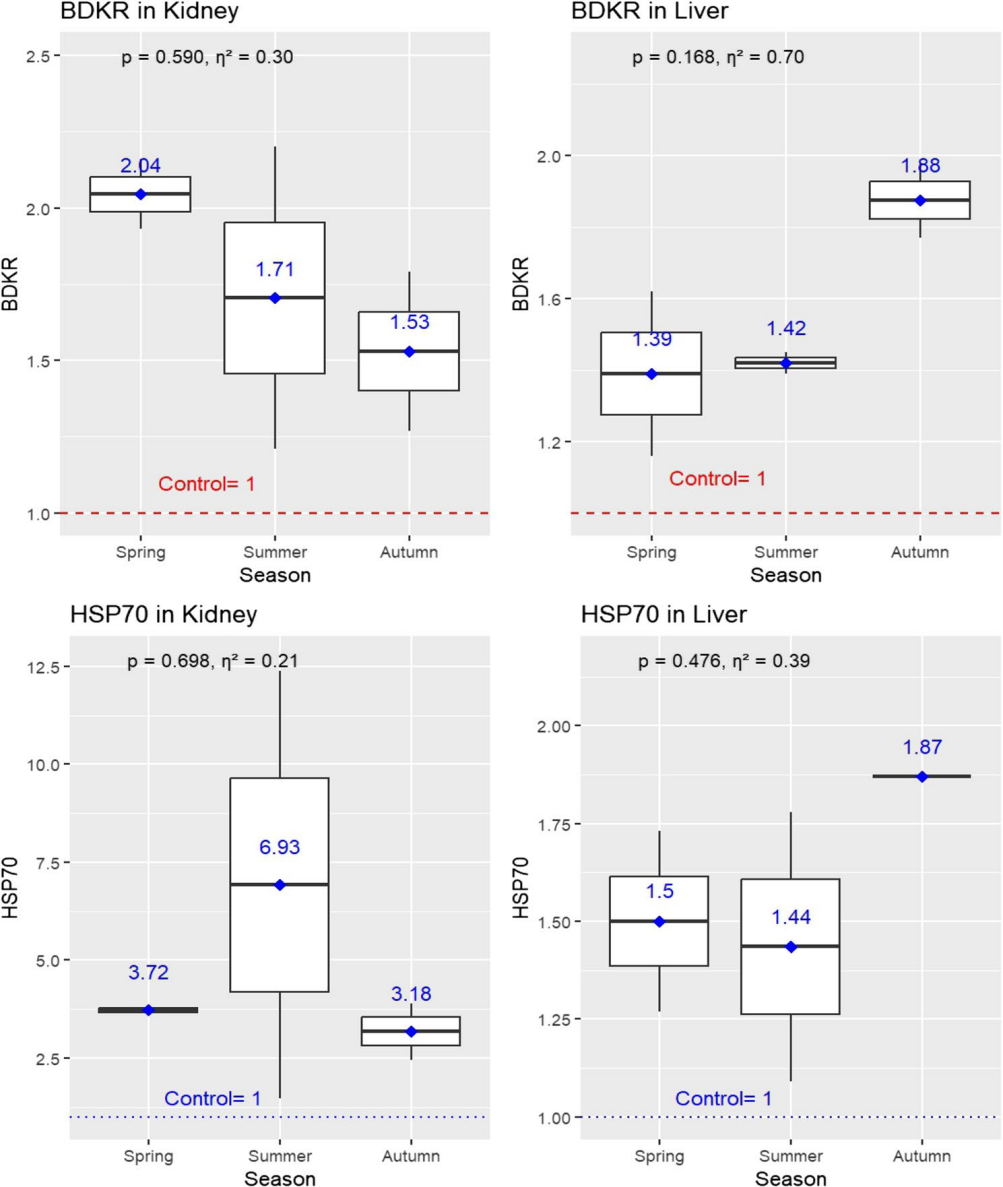
Seasonal variations in the expression levels of BDKR (bradykinin receptor) and HSP70 (heat shock protein 70) genes in kidney and liver tissues of jaundiced sheep carcasses (*n* = 6). Boxplots display gene expression levels across seasons, with mean values annotated above each box. Statistical comparisons were performed using one-way ANOVA with significance set at *p* ≤ 0.05. Effect size analysis (Eta squared η²) was included to assess the magnitude of differences, interpreted as follows: around 0.01 = small, 0.06 = medium, and ≥ 0.14 = large effect

Figure 7 illustrates the expression changes of BDKR and HSP70 genes in the kidney and liver tissues in 16 jaundiced sheep. The BDKR gene in kidney tissues exhibited upregulation during the hot months of spring (2.04 ± 0.16) and summer (1.71 ± 0.70) compared to autumn (1.53 ± 0.37); (*p* = 0.590). Conversely, the liver’s BDKR gene expression was upregulated during the hot months (spring = 1.39 ± 0.33; summer = 1.42 ± 0.04) and showed further elevation in autumn (1.88 ± 0.15; *P* = 0.168). On the other hand, the HSP70 gene in kidney tissues exhibited upregulation during the hot months of spring (3.72 ± 0.16) and summer (6.93 ± 7.71) compared to autumn (3.18 ± 1.02); (*p* = 0.698). While the liver’s HSP70 gene expression was upregulated during the hot months (spring = 1.50 ± 0.33; summer = 1.44 ± 0.49) and showed further elevation in autumn (1.87 ± 0.00; *P* = 0.476).

While none of these seasonal changes reached statistical significance, effect size analysis indicated biologically meaningful trends. For BDKR expression, large effect sizes were observed in both kidney (η² = 0.30) and liver tissues (η² = 0.70). Similarly, HSP70 expression showed large seasonal effects in kidney (η² = 0.21) and liver tissues (η² = 0.39). These findings suggest that seasonal variation, potentially linked to heat stress, may influence the expression of key stress-related genes (Fig. 7).

**Fig. 8:**
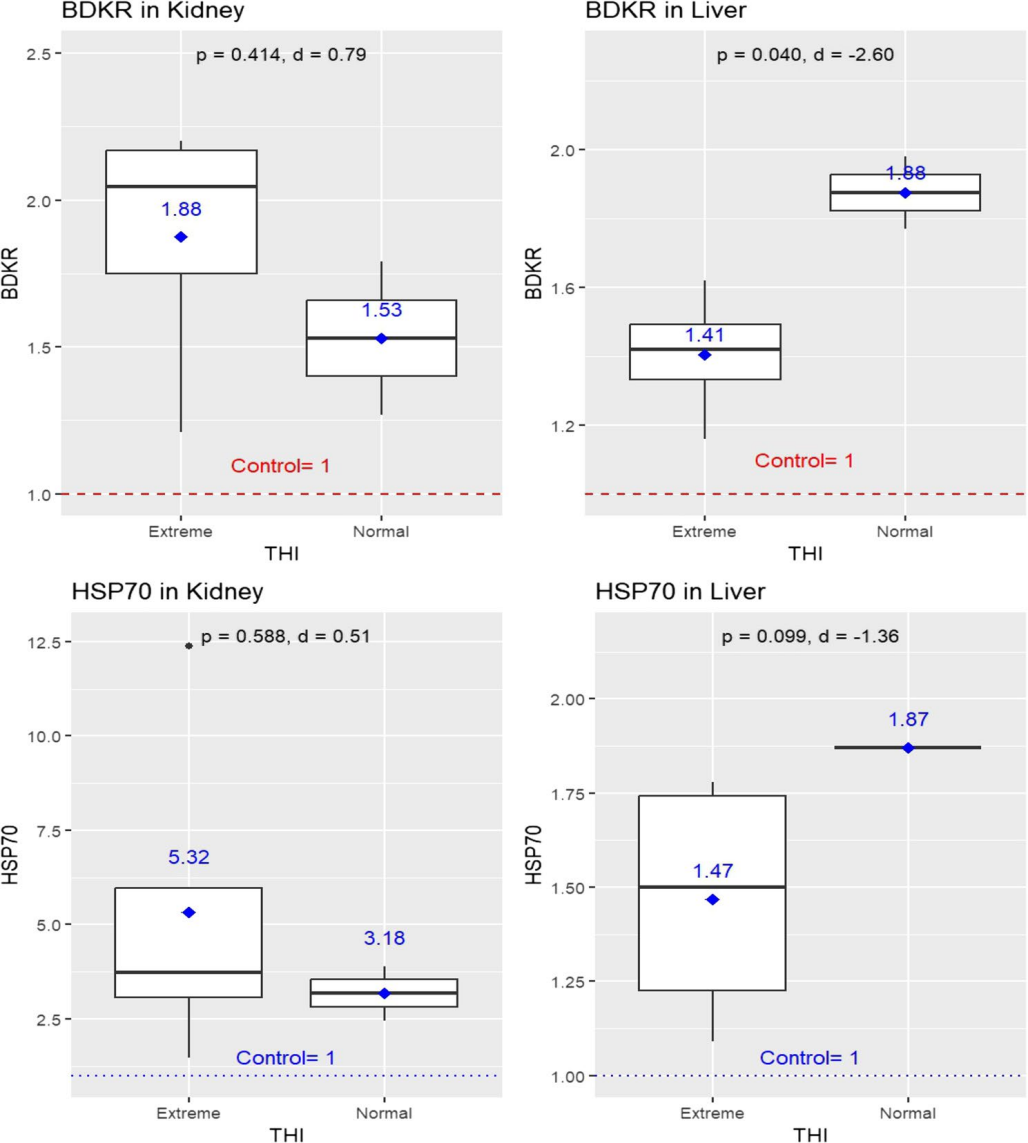
Expression levels of BDKR (bradykinin receptor) and HSP70 (heat shock protein 70) genes in kidney and liver tissues of jaundiced sheep carcasses (*n* = 6) under different heat stress conditions, as defined by the temperature-humidity index (THI). Boxplots show gene expression under extreme (THI > 25.6) and normal (THI < 22.2) conditions, with mean values displayed above each box. Statistical comparisons were performed using independent sample *t*-tests, with significance set at *p* ≤ 0.05. Effect size analysis (Cohen’s *d*) was included to assess the magnitude of differences, interpreted as follows: around 0.2 = small, 0.5 = medium, and ≥ 0.8 = large effect

Moreover, (Fig. 8) showed that extreme THI levels resulted in upregulation of the kidney’s BDKR gene (1.88 ± 0.46) compared to normal THI levels (1.53 ± 0.37); (*p* = 0.414). During extreme THI periods, the liver’s BDKR expression reached a value of 1.41 ± 0.19. Following normalization of THI levels, liver expression significantly increased to 1.88 ± 0.15 (*P* = 0.040). In contrast, extreme THI levels resulted in upregulation of the kidney’s HSP70 gene (5.32 ± 4.82) compared to normal THI levels (3.18 ± 1.02); (*p* = 0.588). In addition, in extreme THI periods, the liver’s HSP70 expression reached a value of 1.47 ± 0.34. Following normalization of THI levels, liver expression increased to 1.87 ± 0.00 (*p* = 0.099).

Despite the lack of statistical significance in most comparisons, effect size analysis revealed biologically meaningful differences. For BDKR expression, a medium effect size was observed in the kidney (Cohen’s *d* = 0.79), while the liver exhibited a large effect (*d* = −2.60), indicating a substantial increase in response to improved thermal conditions. For HSP70 expression, a medium effect was detected in the kidney (*d* = 0.51), and a large effect in the liver (*d* = −1.36), suggesting that hepatic expression of this heat shock protein is particularly responsive to thermal stress modulation. These findings highlight the sensitivity of stress-related gene expression to environmental conditions, underscoring the potential physiological relevance of THI fluctuations (Fig. 8).

### Seasonal and Climatic Temporal patterns of jaundice cases

**Fig. 9:**
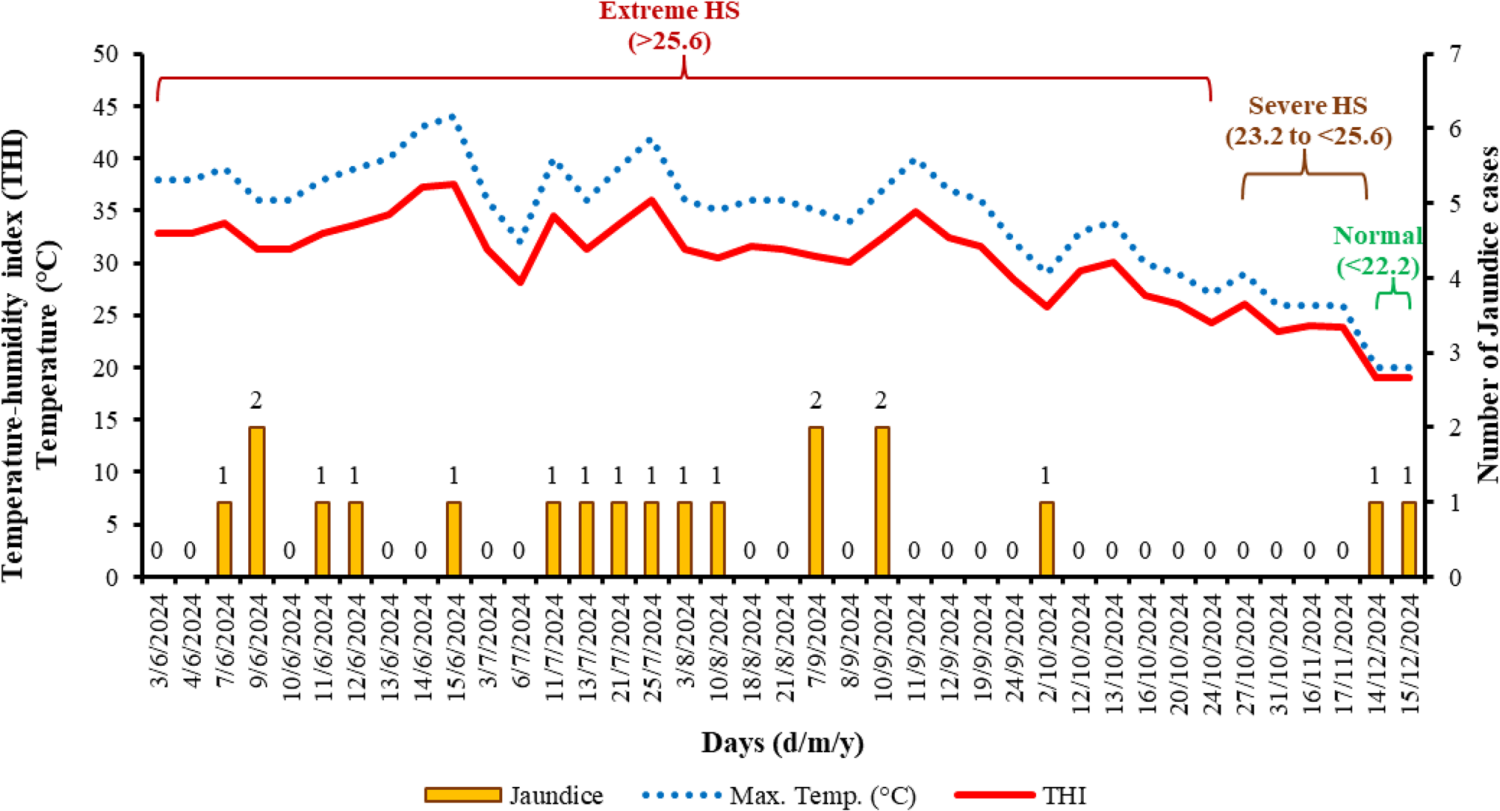
Temporal association between the occurrence of jaundice in sheep carcasses (*n* = 19) and environmental heat stress indicators from June to December 2024. The figure illustrates daily maximum temperature (°C), temperature-humidity index for sheep (THI_sheep_), and categorized heat stress levels (Normal: <22.2, Severe: 23.2–25.6, Extreme: >25.6), in relation to the number of jaundiced carcasses detected per day

Jaundice cases were found to have distinct seasonal and climatic temporal patterns. Throughout the study period, from June to December 2024, the majority of cases were recorded during extreme HS (June, July and September) (Fig. 9). Furthermore, Table 2. shows a significant and strong association between the occurrence of jaundice in sheep and extreme to severe HS conditions, with a Cramer’s coefficient (*V*) ranging from 0.71 to 1.0 (*P* < 0.01). This association is particularly evident during the hotter months (*V* = 0.89 to 1.0; *P* = 0.002 to < 0.0001), and the summer season (*V* = 0.71 to 0.79; *P* = 0.003 to 0.004).

**Table 2:** Association between month, season, and the degree of heat stress on the occurrence of jaundice in sheep carcasses (*N* = 19)

		**THI sheep** **No. of jaundice (%)**		**Estimated RR** **No. of jaundice (%)**			
	**Heat stress level**	**Extreme**	**No Stress**	**Extreme**	**Severe**	**Moderate**	**Normal**
	**Sheep**	17 (89.5)	2 (10.5)	10 (52.6)	6 (31.6)	1 (5.3)	2 (10.5)
**Month**	**June**	6 (31.6)	0	4 (21.1)	2 (10.5)	0	0
	**July**	4 (21.1)	0	4 (21.1)	0	0	0
	**August**	2 (10.5)	0	0	2 (10.5)	0	0
	**September**	4 (21.1)	0	2 (10.5)	2 (10.5)	0	0
	**October**	1 (5.3)	0	0	0	1 (5.3)	0
	**December**	0	2 (10.5)	0	0	0	2 (10.5)
	**Cramer's** ***V***	1.0		0.89			
	***P*** **- value**	0.002*		<0.0001*			
**Season**	**Spring**	6 (31.6)	0	4 (21.1)	2 (10.5)	0	0
	**Summer**	10 (52.6)	0	6 (31.6)	4 (21.1)	0	0
	**Autumn**	1 (5.3)	2 (10.5)	0	0	1 (5.3)	2 (10.5)
	**Cramer's** ***V***	0.79		0.71			
	***P*** **- value**	0.003*		0.004*			

*Asterisk indicates a significant relationship (Cramer's *V*, *P *< 0.05)

*THI* Temperature-Humidity Index, heat stress (HS) categories according to THI values [21]: Absence of HS (< 22.2 units); Moderate HS (22.2 to < 23.2); Severe HS (23.2 to < 25.6); and Extreme HS (>25.6)

*Estimated RR* (RR_est_) Estimated Respiration Rate; the thresholds for RR_est_ categories [22]: Normal = 90; Moderate (alert) = 90-110; Severe (danger) = 110-130; and Extreme (emergency) ≥130

### Climatic variation and shifts in 2024 compared to the previous five years (2019–2023)

**Fig. 10:**
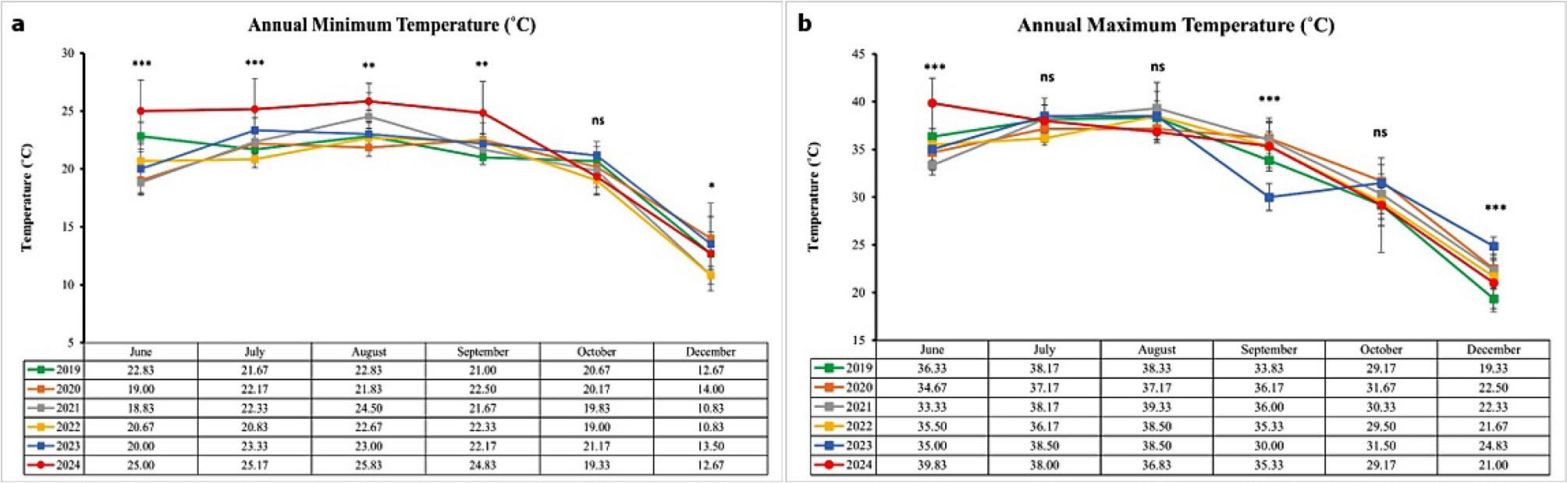
Showing (**a**) minimum and (**b**) maximum temperatures analysis between 2019 and 2024. Asterisks indicate statistical significance levels: *** *p* < 0.001, **p < 0.01,* *p* < 0.05, and ns = not significant

**Fig. 11:**
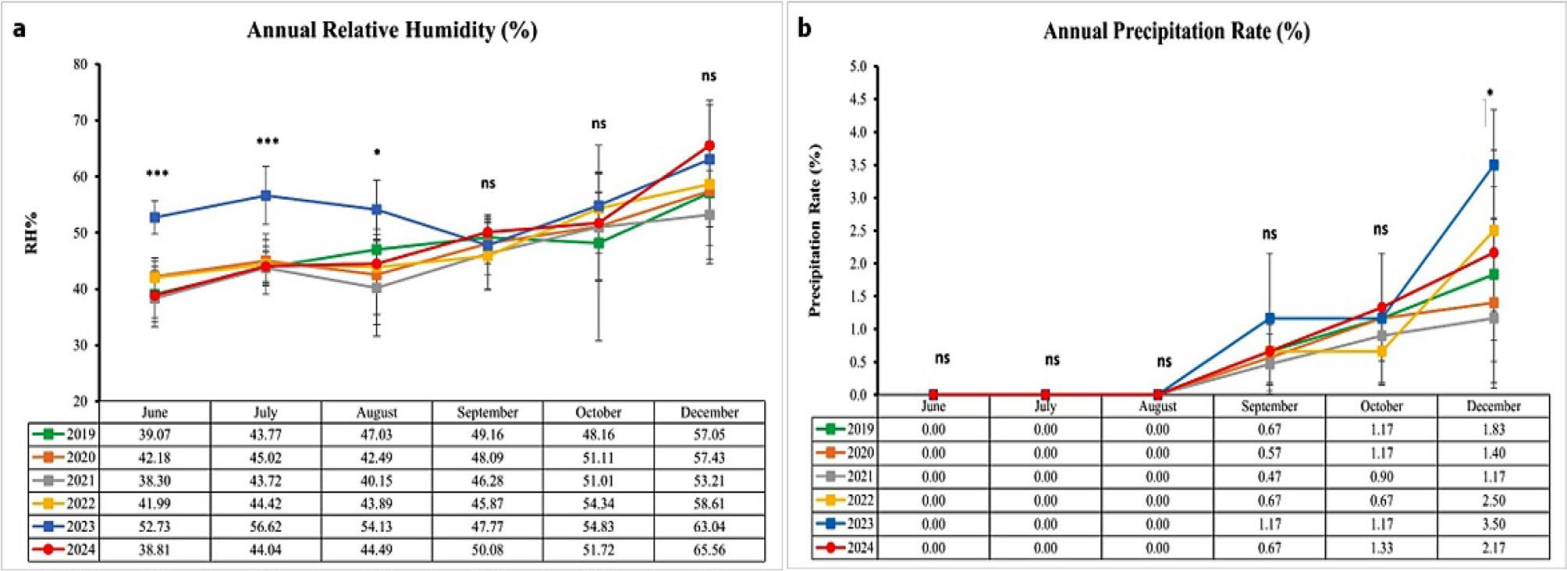
A comparative analysis of climate parameters (**a**) relative humidity, and (**b**) precipitation rate between 2019 and 2024. Asterisks indicate statistical significance levels: *** *p* < 0.001, *p < 0.01, * *p* < 0.05, and ns = not significant

Based on the four climate parameters examined, minimum and maximum temperatures, relative humidity, and precipitation rate, the comparison between 2024 (the year of study) and the preceding five years (2019–2023) indicates clear evidence of climatic shifts. These shifts are particularly evident in increased heat stress, variability in humidity, and subtle changes in rainfall distribution (Figs. 10 and 11).

Temperature shifts revealed that in 2024, minimum temperatures were consistently higher across all studied months (June to December) than in any of the previous years. For instance, in June 2024, the minimum temperature reached 25.0 °C, compared to a range of 18.8 °C to 22.8 °C recorded during 2019–2023. Similar trends were observed in July (25.2 °C) and August (25.8 °C), both representing the highest values for the period. This consistent increase in nighttime temperatures suggests warmer nights and reduced recovery periods, which are critical indicators of cumulative thermal stress in animals (Fig. 10a).

Regarding maximum temperatures, June 2024 recorded 39.8 °C, significantly exceeding the five-year range (33.3 °C to 36.3 °C). Although the maximum temperature in August 2024 (36.8 °C) was slightly lower than in previous years, the overall trend indicates persistent high daytime heat. The elevated diurnal temperature range (difference between daily maximum and minimum temperatures) in 2024 further underscores an intensification of heat load during the warm months (Fig. 10b).

Relative humidity in 2024 remained moderate during the summer (e.g., 38.8% in June and 44.0% in July), slightly lower than the unusually humid summer of 2023 (which reached 52.7% in June and 56.6% in July). However, by December 2024, relative humidity had risen to 65.6%, the highest recorded value over the six-year span. These fluctuations between drier summer and more humid winter conditions may contribute to increased physiological stress in animals and enhanced survival of pathogens (Fig. 11a).

Precipitation during the summer months (June - August) remained absent across all years, indicating a consistent dry season. However, in the autumn and winter months of 2024, rainfall showed a modest increase: September recorded 0.67%, similar to 2022; October reached 1.33%, the highest among all years; and December showed 2.17%, slightly below the 2023 peak (3.50%) but higher than levels recorded in 2019–2021. These findings suggest a gradual shift toward more rainfall in the cooler months, which may influence vector ecology and disease dynamics (Fig. 11b).

In summary, the climate profile of 2024 reflects a clear deviation from historical patterns, with elevated temperatures, increased humidity variability, and shifting rainfall timing. Such changes may exacerbate environmental stressors and facilitate the emergence or amplification of vector-borne diseases such as Rift Valley Fever, which are known to respond to climatic fluctuations.

## Discussion

Climate change significantly impacts the health of both animals and humans [28]. Recent studies identified icterus (jaundice) as a primary cause of total condemnation in sheep. Affected livers were moderately enlarged, soft, and friable, with necrotic patches on the surface. These observations align with findings by Paessler et al., [29]. which also reported necrosis and hemorrhage in the liver, indicating vascular endothelial damage.

Heat stress exacerbated by climate change has been shown to impair vital liver functions such as detoxification and metabolism, potentially leading to liver damage and disruptions in bilirubin metabolism [30].

The histopathological analysis conducted during this study identified centrilobular necrosis and intranuclear inclusion bodies in hepatocytes were identified as hallmark characteristics of RVF in adult sheep. This is consistent with earlier observations by Daubney et al. [31]. which highlighted that hepatocyte infections mostly began in the central zone. Contrarily, other researchers like Nanyingi et al. [32] and Tinto et al. [33] noted these lesions initially occurred more frequently in the midzonal region. Additionally, degenerative changes in hepatocytes were typically accompanied by inflammatory infiltration composed mainly of neutrophils and macrophages. Moreover, distinctive eosinophilic intranuclear inclusions within affected hepatocytes were visibly stained red using the Phloxine-tartrazine staining method [34].

Renal pathology was another significant finding in this study, with multifocal acute renal tubular injury in most cases. Similarly, Daubney et al. [31]. documented acute renal tubular alterations in sheep, showing some tubules to be affected while others appeared unaffected. This study further noted albuminous exudates in Bowman’s capsule, pronounced interstitial edema, and pulmonary congestion. Such changes correspond with observations from Odendaal et al. [35] who linked RVF infections to vascular endothelial damage, along with associated hydropericardium, hydrothorax, ascites, pulmonary congestion, lymphnode edema, and hemorrhages in multiple organs.

Cardiac tissue analysis revealed non-suppurative myocarditis primarily marked by inflammatory cell infiltration between muscle fibers. These findings mirror results by Doosti et al. [36] who reported RVF-associated endothelial infections, necrosis, and mononuclear cell infiltrates within the heart tissue.

Microscopic lung examinations from this study demonstrated necrosis of bronchial epithelium, emphysema, congestion, and interstitial inflammation-paralleling findings by Odendaal et al. [35] where marked interstitial inflammation, emphysema, and sporadic hemorrhages were observed in adult ruminants affected by RVF.

Immunohistochemical analysis highlighted increased expression of Caspase-3 and Tumor Necrosis Factor-alpha (TNF-α) in RVF-affected tissues. These molecules are key mediators of apoptosis and inflammation. Prior research suggested that Caspase-3 upregulation during RVF infection indicates extensive tissue damage and cellular death, particularly in the liver and other target organs [37]. Similarly, TNF-α overexpression critical during inflammatory and immune responses has been associated with systemic inflammation and cytokine storms in severe RVF cases [38]. Climate change exacerbates this immune dysregulation by inducing cellular stress and elevating pro-inflammatory responses such as TNF-α production and activation of apoptotic pathways like those involving Caspase-3 [39]. The broader effects of HS on livestock include compromised growth rates, reduced reproduction and milk production (both quality and quantity), suppressed immunity, and heightened disease susceptibility [40].

The Temperature Humidity Index was used to monitor the intensity of HS; results demonstrated the highest THI values were recorded during June, July, August, and September, aligning with findings by Habeeb et al. [41] who reported Increased THI was strongly associated with extreme HS conditions (THI > 25.6), leading to higher instances of jaundice in sheep. Oxidative stress caused by HS damages liver cells, reducing metabolic efficiency and resulting in bilirubin accumulation, a key factor in jaundice [42].

HS adversely affects the immune system of livestock by impairing immune functions and increasing their vulnerability to diseases [43]. It stimulates the secretion of glucocorticoids, which suppress the production of key pro-inflammatory cytokines such as TNF-α, IL-6, and IL-8. These cytokines are essential for initiating innate immune responses, and their inhibition particularly through the suppression of the p38 MAPK signaling pathway—compromises the stability of the immune system in animals [44]. Moreover, elevated temperatures and increased relative humidity provide optimal conditions for the rapid proliferation of infectious agents and parasites [45]. Climate-induced changes in precipitation and heat load also influence the transmission dynamics of various vector-borne diseases. These include viral diseases (such as lumpy skin disease, Crimean-Congo hemorrhagic fever, RVF, bluetongue, Japanese encephalitis, and bovine ephemeral fever) and bacterial diseases (including pinkeye, coxiellosis, dermatophilosis, anthrax, and leptospirosis) in diverse livestock species [45].

Further analyses confirmed RVF as a causative agent for jaundice in sheep through molecular detection methods involving RVF RNA extraction from liver and kidney samples. This is consistent with studies like those by Azerigyik et al. [8], which reported that climatic extremes influence RVF vector distribution. Rising temperatures were shown to accelerate pathogen incubation periods while enhancing vector reproduction rates, larval survival chances, and overall vector population growth.

Climate change significantly affects the dynamics of vector-borne viral diseases in sheep by disrupting the interactions among the host, pathogen, and environment, the core components of disease ecology [46]. Increased temperatures, shifts in rainfall patterns, and more frequent extreme weather events have been found to enhance the growth, reproduction, and feeding activity of biological vectors like mosquitoes, as climatic conditions have a direct impact on how frequently these vectors feed on their hosts, leading to greater transmission risks of viral diseases such as Rift Valley fever [47]. These environmental disruptions pose major public health threats (SDG 3) and compromise livestock systems, especially in low-resource settings where veterinary infrastructure is limited. Current investigations underscore the profound impact of climate change on livestock health. Liver dysfunction induced by HS or RVF infections highlights how environmental stress exacerbates pathological conditions, further illustrating the intricate links between climate variability and disease proliferation among animals [8].

Recent investigations have observed increased levels of MDA and a decrease in glutathione in kidney and liver samples under extreme temperature-humidity index conditions. This is attributed to HS, which raises the production of free radicals beyond the body’s capacity to neutralize them, resulting in oxidative damage and a reduction in the efficiency of the antioxidant defense system [48, 49]. Furthermore, research by El-Sayed et al. [50] demonstrated that climate-induced stress in sheep impacts the oxidant-antioxidant balance, leading to elevated blood MDA and cortisol levels, while significantly reducing GSH levels. Similarly, El-Tholoth et al. [51] reported that RVF infection increases reactive oxygen species (ROS) production in liver cells due to the presence of the RVF-related protein within mitochondria, which correlates with heightened cytokine and pro-apoptotic gene expressions induced by the infection.

Although the differences in hepatic and renal MDA and GSH levels across seasons and THI conditions did not reach statistical significance, the observed trends are biologically meaningful and suggest a thermally induced shift in redox homeostasis. MDA levels, a reliable index of lipid peroxidation [52], exhibited a consistent increase during hotter months and under extreme THI conditions in both liver and kidney tissues, indicating heightened oxidative challenge. Conversely, GSH concentrations a key marker of intracellular antioxidant defense Fayed et al. [52] showed a trend toward elevation, particularly in kidney tissues during summer and autumn, potentially reflecting an adaptive upregulation of endogenous antioxidant capacity. These directional changes are indicative of a subclinical redox imbalance, likely reflecting early or moderate oxidative stress before the onset of overt damage. Such physiological adaptations are consistent with previous studies reporting that early or moderate HS does not always cause statistically significant oxidative damage but rather initiates a cellular protective response [53].

Heat shock proteins, particularly HSP70, are known to be rapidly induced under thermal stress and serve as molecular chaperones that stabilize protein structure, prevent aggregation, and reduce oxidative burden through modulation of redox-sensitive signaling pathways and participate in the acclimatization [52]. These findings suggest that oxidative stress in the context of HS and RVF infection may manifest in tissue-specific and temporally dynamic ways often detectable at the transcriptional and histopathological level before becoming apparent in gross biochemical changes [54].

Given that sheep are frequently exposed to fluctuating environmental stressors and considering breed-related differences in thermotolerance. Moreover, the observed inter-individual variability and breed-dependent resilience such as the reported lower heat susceptibility in Balkhi versus Damani sheep may have contributed to the lack of statistical significance in oxidative biomarkers, despite consistent directional changes [54]. This supports the view that statistical non-significance should not be equated with biological irrelevance, especially in studies involving small sample sizes and naturally heterogeneous populations.

In this study, gene expression levels of BDKR and HSP70 were found to be upregulated in kidney and liver samples during spring, summer, and autumn. These proteins assist cells in managing various stress types such as heat, cold, oxidative damage, and infection [55, 56]. They protect cells from damage and facilitate their survival and recovery. Samara et al. [55] indicated that thermal stress significantly altered BDKR expression, which is involved in inflammatory responses. Its upregulation implies that HS instigates inflammation and tissue damage, promoting the release of pro-inflammatory cytokines, increased blood vessel permeability, and immune cell migration.

This research also found that HSP70 mRNA expression in response to HS, like earlier findings in sheep [57]. Banerjee et al. [56] noted that HSP70, one of sheep’s most extensively studied heat shock proteins (HSPs), activates due to HS and functions as a molecular chaperone. It aids in protein folding and refolding, prevents protein aggregation, and accelerates the breakdown of damaged proteins. This protein modulates inflammatory responses by interacting with various cytokines such as TNF-α and interleukins.

RVF replication within sheep cells disrupts normal cellular functions, causing an accumulation of misfolded proteins [58]. The cellular stress caused by RVF activation of Heat Shock Factors (HSFs), particularly HSF1, which binds to the heat shock element (HSE) in the promoter region of the HSP70 gene, boosting its transcription and expression [59]. RVF infection triggers the release of pro-inflammatory cytokines like TNF-α, interleukin 6 (IL-6), and type I interferons (IFN-α, IFN-β). These cytokines create a challenging environment for cells, further inducing HSP70 expression as part of the cellular stress response [60]. Several limitations should be acknowledged in the interpretation of this study’s findings: **Sample Size and Statistical Power**: Although biologically relevant trends were observed in oxidative stress markers (MDA, GSH), the relatively small number of jaundiced sheep (*n* = 19) may have limited the statistical power to detect subtle yet meaningful differences.

### Environmental and breed variability

Field conditions involve multiple, uncontrollable variables (e.g., microclimate fluctuations, breed differences in thermotolerance), which may have introduced heterogeneity and masked more consistent responses.

### Lack of functional enzymatic activity assessment

While MDA and GSH levels were measured, the activity of key antioxidant enzymes (e.g., GPX, CAT, SOD) were not assessed. These may have provided further insight into the antioxidant defense system’s functionality under stress.

## Conclusion

This study highlights the intricate link between climate change and livestock health, demonstrating that HS, reflected by elevated temperature-humidity indices, can facilitate the emergence and severity of RVF in sheep. The confirmed presence of RVFV in jaundiced carcasses, along with biochemical indicators of oxidative stress and immune-mediated liver damage, underscores the vulnerability of animals to environmental stressors. These findings stress the importance of adopting climate-resilient livestock management practices, enhancing disease surveillance, and improving public health preparedness. Addressing these challenges directly supports the United Nations Sustainable Development Goals, particularly SDG 2 (Zero Hunger), SDG 3 (Good Health and Well-being), SDG 13 (Climate Action), and SDG 15 (Life on Land), by promoting sustainable food systems, animal welfare, and ecosystem resilience.

## Data Availability

The research data used to support the findings of this study are included within the article.

## Abbreviations

RVFV: Rift Valley Fever Virus
RVF: Rift Valley fever
HS: Heat stress
TNFα: Tumor necrosis factor alpha
HSP70: Heat shock 70KDa protein 1A
BDKRB1: Bradykinin receptor B1
MDA: Malondialdehyde
GSH: Reduced glutathione
ACTB: Actin beta
THI: Temperature Humidity Index
